# Mechanistic Insight into the Elastin Degradation Process by the Metalloprotease Myroilysin from the Deep-Sea Bacterium *Myroides profundi* D25

**DOI:** 10.3390/md13031481

**Published:** 2015-03-18

**Authors:** Jie Yang, Hui-Lin Zhao, Bai-Lu Tang, Xiu-Lan Chen, Hai-Nan Su, Xi-Ying Zhang, Xiao-Yan Song, Bai-Cheng Zhou, Bin-Bin Xie, Anthony S. Weiss, Yu-Zhong Zhang

**Affiliations:** 1 State Key Laboratory of Microbial Technology, Marine Biotechnology Research Center, Shandong University, Jinan 250100, China; E-Mails: yangjie199102@163.com (J.Y.); zhaohuilin1984@163.com (H.-L.Z.); tangbailu@yeah.net (B.-L.T.); cxl0423@sdu.edu.cn (X.-L.C.); suhn@sdu.edu.cn (H.-N.S.); zhangxiying@sdu.edu.cn (X.-Y.Z.); xysong@sdu.edu.cn (X.-Y.S.); bczhou@qdio.ac.cn (B.-C.Z.); zhangyz@sdu.edu.cn (Y.-Z.Z.); 2 Marine Biotechnology Research Center, Shandong University, Jinan 250100, China; 3 Department of Pathogenic Biology, Binzhou Medical University, Shandong Province, Yantai 264003, China; 4 School of Molecular Bioscience, The University of Sydney, NSW 2006, Australia; E-Mail: tony.weiss@sydney.edu.au

**Keywords:** deep sea, elastase, bacteria, degradation mechanism, biotechnological potential

## Abstract

Elastases have been widely studied because of their important uses as medicine and meat tenderizers. However, there are relatively few studies on marine elastases. Myroilysin, secreted by *Myroides profundi* D25 from deep-sea sediment, is a novel elastase. In this study, we examined the elastin degradation mechanism of myroilysin. When mixed with insoluble bovine elastin, myroilysin bound hydrophobically, suggesting that this elastase may interact with the hydrophobic domains of elastin. Consistent with this, analysis of the cleavage pattern of myroilysin on bovine elastin and recombinant tropoelastin revealed that myroilysin preferentially cleaves peptide bonds with hydrophobic residues at the P1 and/or P1′ positions. Scanning electron microscopy (SEM) of cross-linked recombinant tropoelastin degraded by myroilysin showed preferential damages of spherules over cross-links, as expected for a hydrophobic preference. The degradation process of myroilysin on bovine elastin fibres was followed by light microscopy and SEM, revealing that degradation begins with the formation of crevices and cavities at the fibre surface, with these openings increasing in number and size until the fibre breaks into small pieces, which are subsequently fragmented. Our results are helpful for developing biotechnological applications for myroilysin.

## 1. Introduction

Elastin, an important protein in all higher organisms, including marine animals, is highly cross-linked, water-insoluble and persistent and is therefore resistant to both chemical and biological degradation [1,2]. As a result, most elastin in the sea likely deposits into sediments and becomes an important constituent of sedimentary particulate organic nitrogen (PON) [3]. Elastin is fundamentally composed of tropoelastin [4,5,6], and two major types of domains, hydrophobic and hydrophilic, are arranged alternately in the tropoelastin sequence. The hydrophobic domains are rich in nonpolar residues and are involved in the alignment of tropoelastin, whereas the hydrophilic domains are mostly composed of Lys and Ala and participate in cross-linking [2,7]. The process of insoluble elastin formation mainly includes the coacervation of ~15-nm soluble tropoelastin molecules into micron-sized spherules and lysyl oxidase-mediated cross-linking of these spherules [1,8,9]. Following complex cross-linking, the insoluble elastin fibres are resistant to most proteases and, thus, only sensitive to a limited number of elastases.

Elastases are a type of protease capable of solubilizing fibrous elastin. Because of their ability to regulate lipid metabolism, reduce blood cholesterol and remove denatured elastins on atherosclerotic plaques, elastases have been used to effectively treat hyperlipidaemia and to prevent atherosclerosis [10,11]. In addition, because elastases in tissues are often related to cigarette smoking-induced disease in human, such as pulmonary emphysema, these enzymes are utilized to develop experimental models of cigarette smoking-induced diseases *in vitro* for laboratory research [12]. In addition, elastases are applied as ideal meat tenderizers in the food industry due to their specific degradation of connective tissues [13]. To date, detailed investigation and development of elastases have mainly involved those isolated from terrestrial mammals. Regardless, several marine bacterial elastases have been reported [3,14], indicating that marine bacteria are likely a good resource for discovering new elastases for biotechnological use.

*Myroides profundi* D25 is a protease-producing bacterium that we isolated from the deep-sea sediment near the southern Okinawa Trough [15]. Strain D25 secretes a novel astacin-like metalloprotease, myroilysin, which is the most abundant protease secreted by this strain. Myroilysin is an elastase with an optimum temperature of 40 °C and an optimum pH of 9.0; Zn^2+^ can severely inhibit the elastin-degrading activity of myroilysin. In addition to its high elastin-degrading activity, myroilysin also has strong collagen-swelling ability and plays a synergistic role with collagenase in collagen hydrolysis [3]. These characteristics of myroilysin suggest that this bacterial elastase may have promising biotechnological applications.

In this study, we investigated the elastin-degrading mechanism of myroilysin using bovine elastin and recombinant tropoelastin as substrates, and we found that myroilysin molecules bind hydrophobically to insoluble bovine elastin fibres. The elastin degradation mechanism of myroilysin was then assessed by analysing cleavage sites on bovine elastin and observing the degradation process using light microscopy (LM) and scanning electron microscopy (SEM). We further explored the myroilysin-catalysed elastolysis of recombinant tropoelastin by observing the degradation process of cross-linked tropoelastin and analysing cleavage sites.

## 2. Results

### 2.1. Hydrophobic Binding of Myroilysin to Insoluble Elastin

**Figure 1:**
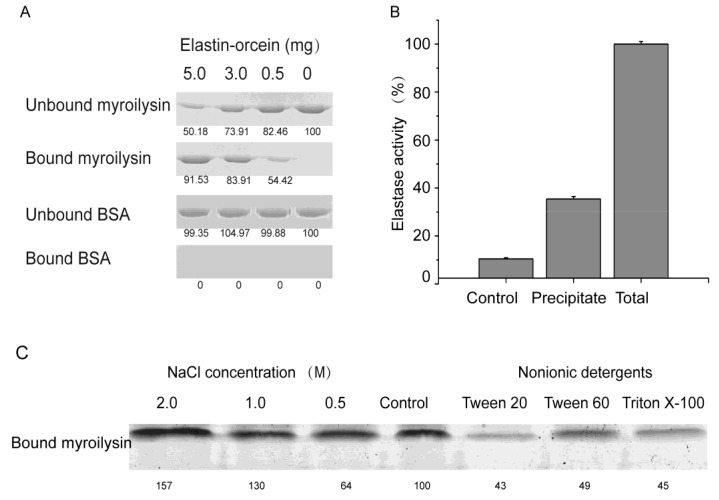
Binding of myroilysin to insoluble elastin fibres through hydrophobic interaction. (**A**) SDS-PAGE analysis of the ability of myroilysin to bind to insoluble elastin-orcein. Bovine serum albumin in place of myroilysin was used as a negative control. The bound and unbound fractions were analysed by 12.5% SDS-PAGE. The numbers at the bottom of the gel are the densitometric ratios of each band compared with that of the control band. (**B**) The elastin-degrading activity of myroilysin bound to insoluble elastin-orcein. The total activity of 0.25 mL myroilysin solution was taken as 100%. The activity of the mixture of 5 mg elastin-orcein with buffer served as a control. The data are from three experimental repeats (mean ± S.D.). (**C**) Effects of NaCl and nonionic detergents on the binding of myroilysin to insoluble elastin-orcein.

Proteases that degrade insoluble proteins initially bind to their insoluble substrates [16,17,18]. Our previous work showed that myroilysin has a high activity towards insoluble elastin [3]. To investigate whether myroilysin can bind to insoluble elastin, the activity of myroilysin was first inhibited with exogenous Zn^2+^, and the elastin-binding ability of myroilysin was then assessed by SDS-PAGE. As shown in Figure 1A, the amount of bound myroilysin increased with increasing elastin-orcein concentration, consistent with myroilysin binding to insoluble elastin. Moreover, when the exogenous Zn^2+^ was removed, significant degradation of the treated elastin-orcein was detected after incubation (Figure 1B), indicating that the myroilysin molecules that bound to elastin during the mixing step degraded the substrate after the removal of exogenous Zn^2+^. Taken together, these results demonstrated that myroilysin has the ability to bind to insoluble elastin.

To further explore the binding of myroilysin to insoluble elastin, we examined the effects of NaCl and nonionic detergents. As shown in Figure 1C, NaCl at low concentrations had little effect on the binding of myroilysin to insoluble elastin, whereas NaCl at the high concentration of 2 M increased its binding to the fibres. In contrast, three nonionic detergents, Tween 20, Tween 60 and Triton X-100, all significantly decreased enzyme binding. These results indicated that myroilysin binds to insoluble elastin mainly through hydrophobic interactions.

### 2.2. Cleavage Sites of Myroilysin in Bovine Elastin

**Figure 2:**
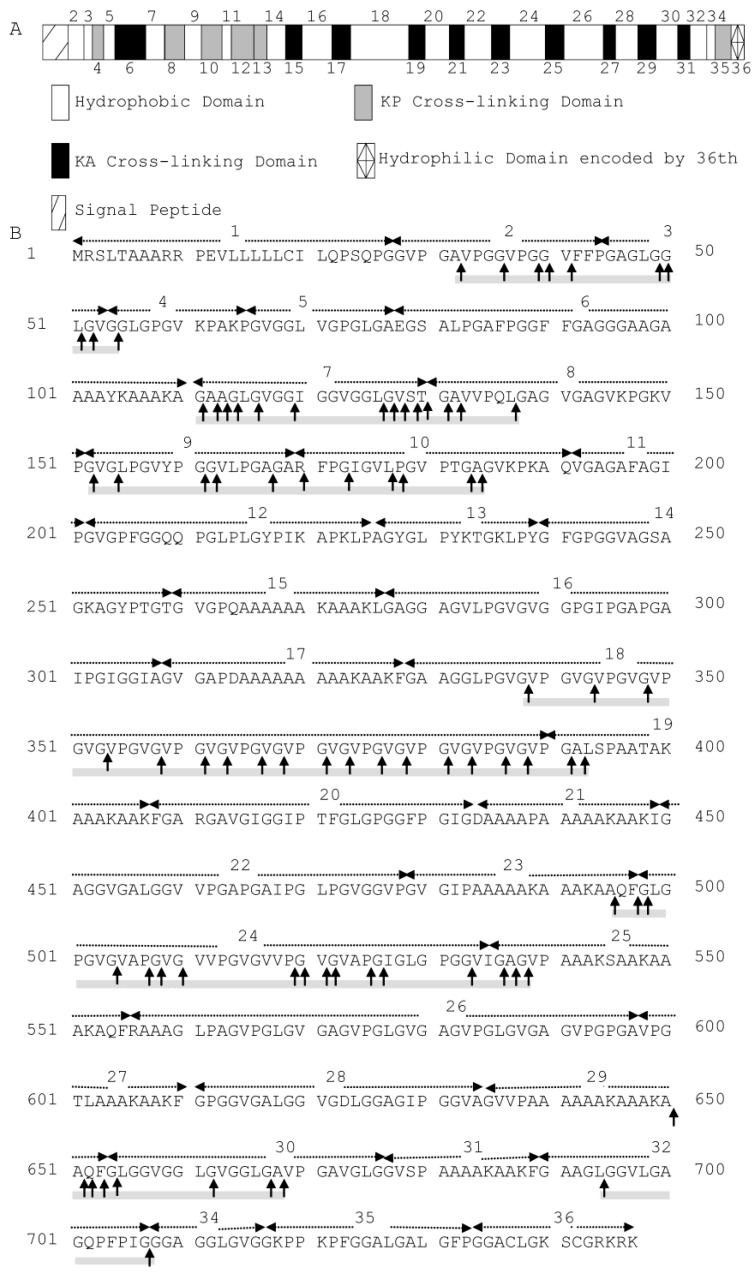
(**A**) Domain map of bovine tropoelastin containing all possible exons. The hydrophilic cross-linking domains are further divided into KP cross-linking domains, with lysine pairs separated by one or more proline residues, and KA cross-linking domains, with lysine pairs separated by alanine residues. Domain 36 is assigned differently because of its unique structural features [19]. (**B**) Cleavage sites of myroilysin in bovine tropoelastin (SwissProt Accession Number P04985-1). Each dashed arrow above the sequence indicates a domain encoded by an individual exon. The cleavage sites are marked by vertical arrows. The cleavage sites were determined based on the sequences of peptides from bovine elastin released by myroilysin, as shown in Supplementary Table S1. The sequences covered by the determined peptides are underlined with grey solid lines.

Bovine elastin predominantly comprises cross-linked bovine tropoelastin, which is composed of multiple hydrophobic and hydrophilic domains, typically arranged in an alternating fashion. These domains are encoded by different exons, with the hydrophobic domains encoded by exons 2, 3, 5, 7, 9, 11, 14, 16, 18, 20, 22, 24, 26, 28, 30, 32 and 34 [19] (Figure 2A). The cleavage sites of myroilysin on bovine elastin were analysed by LC-MS. Based on the sequences of 93 released peptides that were identified (Supplementary Table S1), 81 possible cleavage sites were determined; these sites are marked by arrows on the sequence of bovine tropoelastin shown in Figure 2B. Because the sequence VGVPG repeats many times in the domain encoded by exon 18, all of the possible cleavage sites on bovine tropoelastin deduced from the identified peptide sequences VPGVGVPGVGVPGVGVPGVGVPGVGVPGVG, VPGVGVPGVGVPGVG, VPGVGVPGVGVPGVGVPGVGVPG, VPGVGVPGVGVPGVGVPGVGVPGVG are shown in Figure 2B. Elastin largely consists of the hydrophobic amino acid residues Gly, Val, Ala and Pro, which account for up to 75% of its total sequence, among which approximately 33% is Gly [9]. Of the 81 myroilysin recognition sites on elastin, Gly had the highest occurrence in each site from P4 through to P4′, likely because Gly is the most abundant residue in elastin. In addition, more than 90% of the P1′ positions were occupied by hydrophobic residues, including Gly (25.93%), Val (43.21%), Leu (7.41%), Ala (9.88%), Ile (3.70%), Pro (1.23%) and Phe (3.70%). The P1 position of these cleavage sites was also primarily occupied by hydrophobic amino acid residues: Gly (64.20%), Ala (14.81%), Val (3.70%), Leu (4.94%), Pro (4.94%) and Phe (2.47) (Table 1). In contrast, the proportion of hydrophilic amino acid residues at the P1′ or P1 position was very low. Therefore, myroilysin shows preferential cleavage of peptide bonds with hydrophobic residues at the P1′ and/or P1 positions. Moreover, the myroilysin cleavage sites in bovine elastin were mainly in the hydrophobic domains encoded by exons 2, 3, 7, 9, 18, 24 and 30, with only a few cleavage sites observed in the hydrophilic cross-linking domains (Figure 2B). These results indicate that myroilysin preferentially digests the hydrophobic domains of bovine elastin.

**Table 1:** Myroilysin specificity for bovine elastin ^a^.

	P4	P3	P2	P1	P1′	P2′	P3′	P4′	Occurrence Number
G	30.86	37.04	23.46	64.20	25.93	32.10	46.91	22.22	229
V	17.28	20.99	23.46	3.70	43.21	17.28	19.75	27.16	140
P	20.99	13.58	17.28	4.94	1.23	20.99	12.35	22.22	92
A	14.81	11.11	13.58	14.81	9.88	7.41	4.94	9.88	70
L	4.94	3.70	8.64	4.94	7.41	9.88	6.17	8.64	44
Y	1.23	1.23	0.00	0.00	0.00	0.00	0.00	0.00	2
F	1.23	1.23	2.47	2.47	3.70	4.94	3.70	2.47	18
I	2.47	1.23	2.47	0.00	3.70	2.47	0.00	2.47	12
K	3.70	3.70	1.23	0.00	0.00	0.00	1.23	1.23	9
Q	0.00	2.47	3.70	1.23	2.47	1.23	0.00	1.23	10
T	0.00	2.47	2.47	1.23	1.23	1.23	1.23	2.47	10
R	1.23	0.00	0.00	1.23	0.00	0.00	1.23	0.00	3
S	1.23	1.23	1.23	1.23	1.23	2.47	2.47	0.00	9

^a^ Occurrence of different amino acids at the substrate positions P1–P4 and P1′–P4′, which refers to the preference for a particular amino acid at a given position. The last column shows the number of occurrences of each amino acid.

**Figure 3:**
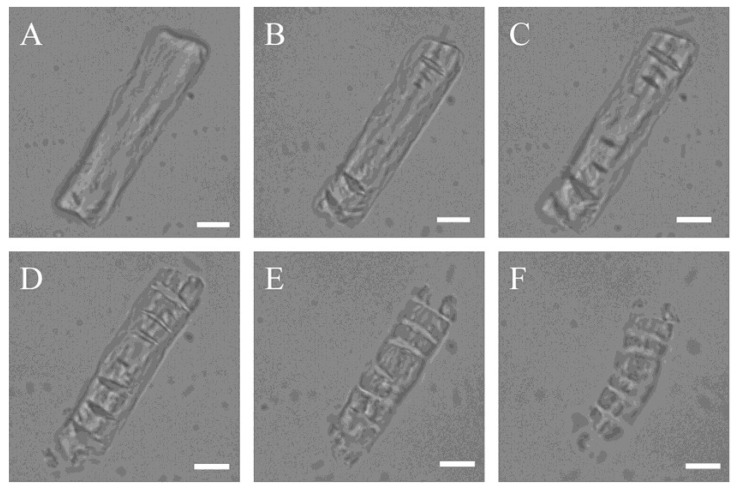
*In situ* observation of the degradation of a bovine elastic fibre by myroilysin: (**A**) 0 min; (**B**) 5 min; (**C**) 10 min; (**D**) 15 min; (**E**) 20 min; (**F**) 25 min. Magnification is ×960. Bars: 5 μm.

### 2.3. LM and SEM Observations of Bovine Elastin Fibre Degradation by Myroilysin

Using LM, it has been found that myroilysin cut insoluble elastin fibres into fragments [3]. In the present study, we further observed the degradation process of one elastin fibre by myroilysin *in situ* under LM. The untreated elastic fibre exhibited an unbroken and smooth surface (Figure 3A). However, after 5 min of treatment with myroilysin at room temperature, transverse crevices began to appear on both ends (Figure 3B), increasing in number and size over time (Figure 3C), until the material was lost from both ends of the elastin fibre (Figure 3D,E). The dissociated material was degraded further by myroilysin and subsequently disappeared from the field of view (Figure 3E,F). These *in situ* observations corroborated our previous work that myroilysin degrades elastin fibres transversely [3].

**Figure 4:**
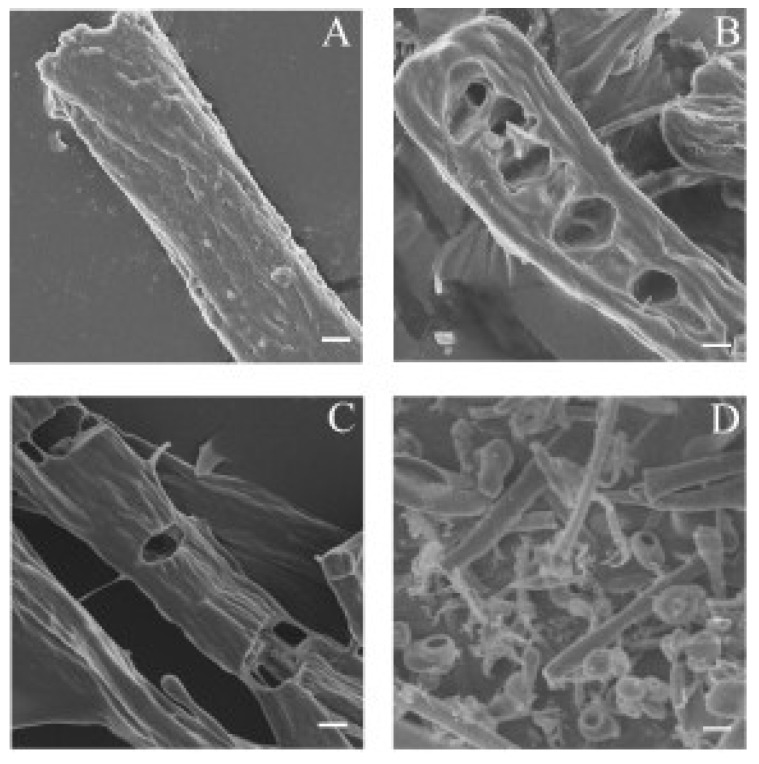
SEM of bovine elastin fibre degradation by myroilysin. A mixture of 0.2 mL myroilysin (0.05 mg/mL) with 5 mg bovine elastin fibres in 50 mM Tris-HCl buffer (pH 9.0) was incubated at 37 °C with continuous stirring. The same reaction system without myroilysin served as a control (**A**). At different time points of digestion ((**B**) 30 min; (**C**) 60 min; (**D**) 120 min), the elastin fibres were separated and washed twice with deionized water. After lyophilization, the samples were mounted on a metal grid and coated with 5 nm platinum prior to SEM at 5.0 kV. Bars: 2 μm.

The time-dependent progressive disintegration of elastin fibres by myroilysin was further examined by SEM. Under SEM, elastin fibres are seen as fibrils arranged in parallel with diameters ranging from 6 to 10 μm (Figure 4A). After 30 min of incubation with myroilysin, cavities appeared on the surface of the elastic fibres (Figure 4B), which then gradually enlarged in depth and width (Figure 4C). After 2 h of digestion, the intact elastin fibres were completely deformed, leaving spherules and fibril fragments in the field of view (Figure 4D).

Taken together, our LM and SEM observations and the above results established that myroilysin initially binds and degrades hydrophobic domains on the surface of elastin fibres.

### 2.4. SEM Observation of the Degradation of Cross-Linked Recombinant Tropoelastin Spherules by Myroilysin

**Figure 5:**
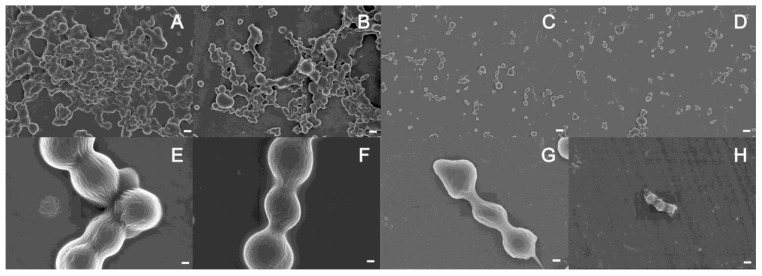
Degradation of recombinant tropoelastin cross-linking clusters by myroilysin. Tropoelastin was cross-linked with BS3 then incubated with myroilysin for: 0 min (**A**,**E**); 20 min (**B**,**F**); 60 min (**C**,**G**) and 150 min (**D**,**H**). **E**–**H** are the same samples as **A**–**D**, respectively, with higher magnification. Bars in **A**–**D**: 2μm. Bars in **E**–**H**: 300 nm.

Cross-linked tropoelastin was selected as a simple elastin analogue that conveniently lacks complex maturation. The hydrophobic domains promote the association of tropoelastin by coacervation to form spherules, whereas the hydrophilic domains are involved in forming cross-links [2,7]. We prepared cross-linked tropoelastin on this basis, and tropoelastin spherules and the cross-links connecting these spherules were both clearly observed under SEM (Figure 5A,E). After treatment with myroilysin for 20 min, the cross-links between the spherules were retained, but the shape of the spherules began to distort, indicating that myroilysin was degrading the spherules (Figure 5B,F). With continued treatment, all of the spherules in the field of view appeared to be degraded and became smaller. After 60 min of treatment, the smaller spherules had almost disappeared, while the larger ones became ovoid due to further degradation by myroilysin (Figure 5C,G). After 150 min of treatment, most spherules had disappeared, and only some irregular cross-linked fragments remained (Figure 5D,H).

### 2.5. Cleavage Sites of Myroilysin in Recombinant Tropoelastin

**Figure 6:**
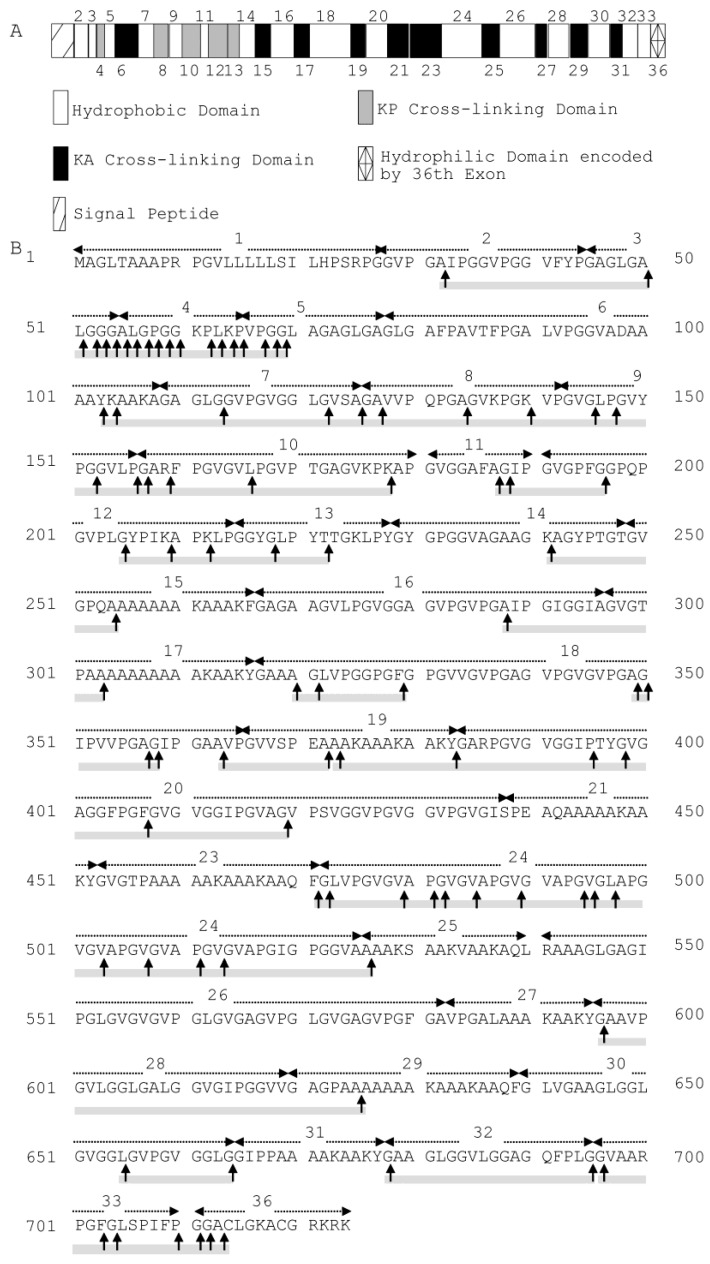
(**A**) Domain map of human tropoelastin containing all possible exons. The hydrophilic cross-linking domains are further divided into KP cross-linking domains, with lysine pairs separated by one or more proline residues, and KA cross-linking domains, with lysine pairs separated by alanine residues. Domain 36 is assigned differently because of its unique structural features [2]. (**B**) Cleavage sites of myroilysin in recombinant human tropoelastin. Each dashed arrow above the sequence indicates a domain encoded by an individual exon. The cleavage sites are marked by vertical arrows. The cleavage sites were determined based on the sequences of peptides from recombinant human tropoelastin released by myroilysin, as shown in Supplementary Table S2. The sequences covered by the determined peptides are underlined with grey solid lines.

The myroilysin cleavage sites in recombinant tropoelastin were further analysed. Based on the sequences of 101 released peptides that were identified (Supplementary Table S2), 107 possible cleavage sites were determined; these sites are marked by arrows on the sequence of human tropoelastin shown in Figure 6B. Because the identified peptide sequence APGVGVAPGV has four repeats in the domain encoded by exon 24, and GVPGVGGLG appears in both domains encoded by exons 7 and 30, all off the possible cleavage sites deduced by these sequences on human tropoelastin are shown in Figure 6B. Among the cleavage sites, more than 90% of the P1′ positions were occupied by hydrophobic amino acid residues, including Gly (36.67%), Val (12.22%), Pro (3.33%), Ala (22.22%), Leu (11.11%), Ile (5.56%), Tyr (1.11%) and Phe (1.11%). The P1 position of these cleavage sites was also largely occupied by hydrophobic amino acid residues: Gly (36.67%), Val (7.78%), Pro (11.11%), Ala (21.11%), Leu (6.67%), Tyr (1.11%) and Phe (1.11%) (Table 2). Except for Lys, which occurred at the P1 (7.78%) and P1′ (3.33%) positions with in moderate proportion, the proportion of other hydrophilic residues at the P1′ or P1 position was very low: Thr (2.22%) at P1′; Thr (1.11%) and Arg (1.11%) at P1. Therefore, myroilysin also preferentially cleaves peptide bonds of recombinant tropoelastin with hydrophobic residues at the P1′ and/or P1 positions. In addition to hydrophobic domains, some cleavage sites were also distributed in the hydrophilic domains of recombinant tropoelastin (Figure 6B).

**Table 2:** Myroilysin specificity for recombinant human tropoelastin ^a^.

	P4	P3	P2	P1	P1′	P2′	P3′	P4′	Occurrence Number
G	28.89	22.22	35.56	36.67	36.67	16.67	31.11	22.22	207
V	14.44	13.33	3.33	7.78	12.22	16.67	8.89	22.22	89
P	14.44	31.11	8.89	11.11	3.33	27.78	15.56	20.00	119
A	20.00	14.44	17.78	21.11	22.22	17.78	17.78	14.44	131
L	5.56	6.67	10.00	6.67	11.11	8.89	6.67	6.67	56
Y	3.33	0.00	7.78	2.22	1.11	1.11	3.33	0.00	17
F	3.33	2.22	5.56	4.44	1.11	0.00	1.11	1.11	17
I	1.11	1.11	3.33	0.00	5.56	3.33	1.11	2.22	16
C	0.00	0.00	0.00	0.00	1.11	1.11	1.11	1.11	4
K	3.33	5.56	3.33	7.78	3.33	3.33	7.78	5.56	36
Q	1.11	1.11	2.22	0.00	0.00	0.00	1.11	1.11	6
T	1.11	1.11	0.00	1.11	2.22	0.00	0.00	1.11	6
R	1.11	0.00	0.00	1.11	0.00	1.11	2.22	1.11	6
S	2.22	0.00	1.11	0.00	0.00	2.22	2.22	1.11	8
E	0.00	1.11	1.11	0.00	0.00	0.00	0.00	0.00	2

^a^ Occurrence of different amino acids at the substrate positions P1–P4 and P1′–P4′, which refers to the preference for a particular amino acid at a given position. The last column shows the number of occurrences of each amino acid.

## 3. Discussion

The majority of studied and applied elastases are those from terrestrial animals and non-marine bacteria. In contrast, there are only a few studies focusing on the elastases from marine organisms. Nonetheless, marine elastases are most likely various and abundant because of the large number of marine animals containing elastin. Therefore, it is important to study marine elastases to develop new elastases and applications. In recent years, we have described two marine bacterial elastases: myroilysin from *Myroides profundi* D25 [3] and pseudoalterin from *Pseudoalteromonas* sp. CF6-2 [14]. Myroilysin is an enzyme of the M12 family, and pseudoalterin is a member of the M23 family. Under LM, myroilysin and pseudoalterin display distinct degradation patterns on insoluble elastin fibres, with myroilysin cutting the elastin fibre into pieces and pseudoalterin releasing filaments from the elastin fibre. We have reported the elastin-degrading mechanism of pseudoalterin, which hydrolyzes peptide bonds in elastin cross-linking domains prior to the degradation of Gly-Xaa bonds. Therefore, morphologically, pseudoalterin first releases elastin filaments from an elastin fibre by breaking down cross-links in the fibre [14]. In this study, we present the elastin-degrading mechanism of myroilysin, which is complementary to that of pseudoalterin.

In recent years, significant progress has been made on the assembly and structure of elastin. The assembly of elastin fibre relies on its dominant component, tropoelastin. Recent studies showed that a tropoelastin molecule consists of an elastin coil region and a cell-interactive C-terminal foot region, which are connected by a highly exposed bridge region [20,21]. A head-to-tail model for elastin assembly by tropoelastin was proposed [20]. In this model, juxtaposed domains 19 and 25 on one tropoelastin molecule are cross-linked to domain 10 of an adjacent tropoelastin molecule to form desmosine. This assembly results in covalently-bonded tandem molecules to yield polymers with a diameter of approximately 5–8 nm, which are further cross-linked to form more complicated structures. The lateral association between bonded molecules is driven by hydrophobic interactions [20]. Based on these studies, it is possible that the surfaces of the filaments in a cross-linked elastin fibre are covered with hydrophobic domains and that the hydrophilic domains are mainly distributed among filaments at sites where they are primarily devoted to cross-linking.

We studied the degradation of bovine elastin fibres by myroilysin using chemical analysis and microscopic observation. The results revealed that after hydrophobically binding to the surface of an elastin fibre, myroilysin begins to degrade the hydrophobic domains on the fibre surface, leading to the formation of crevices and cavities on the fibre surface, with degradation continuing through stepwise fragmentation. SEM observation of cross-linked recombinant tropoelastin also indicated that myroilysin preferentially degrades hydrophobic domains. This elastin-degrading mechanism of myroilysin is very different from that of pseudoalterin, which preferentially cleaves bonds around cross-links to release filaments from an elastic fibre [14].

The cleavage sites of myroilysin on bovine elastin and recombinant tropoelastin were analysed by LC-MS. However, due to the limitation of this method, only a portion of the cleavage sites could be determined. According to the determined sites, myroilysin preferentially cleaves peptide bonds of bovine elastin and recombinant tropoelastin with hydrophobic residues at the P1 and/or P1′ positions. The cleavage sites were found to be mainly distributed in the hydrophobic domains in bovine elastin, likely because the cross-link regions in bovine elastin are protected from lysis by hydrophobic domains, prohibiting access by the enzyme. In contrast, the myroilysin cleavage sites in recombinant tropoelastin are also distributed in hydrophilic domains in addition to hydrophobic domains. Several matrix metalloproteinases (MMPs) have been shown to have elastolytic activity, such as MMP-7, MMP-9 and MMP-12 [22,23]. Heinz *et al.* reported the cleavage site specificities of these three enzymes on recombinant tropoelastin, with these MMPs showing a strong preference for Leu at P1′ and Pro at P3 and tolerating hydrophobic and/or aliphatic amino acids, including Pro, Gly, Ile and Val, at P1′ [22]. Our results showed that myroilysin has a cleavage specificity for tropoelastin different from these MMPs.

## 4. Experimental Section

### 4.1. Reagents and Materials

Recombinant tropoelastin encoded by the synthetic human tropoelastin gene (SHELdelta26A) was prepared as previously described [24,25]. The cross-linking agent bis(sulfosuccinimidyl)suberate (BS3), elastin from bovine neck ligament (SwissProt Accession Number P04985-1) and bovine serum albumin (BSA) were purchased from Sigma (St. Louis, MO, USA). Myroilysin was purified as previously described [3]. BCA assays (Pierce, Rockford, IL, USA) were used to determine protein concentrations.

### 4.2. Assay of the Ability of Myroilysin to Bind to Insoluble Elastin

The ability of myroilysin to bind to insoluble elastin-orcein was analysed using the method of Valenzuela *et al.* [26], with minor changes. Myroilysin (0.2 mg/mL) was first dissolved in 50 mM Tris-HCl (pH 9.0) containing 1 mM Zn^2+^ (Buffer A) to inhibit its elastin-degrading activity. Then, 0.2 mL of enzyme solution were mixed with 5, 3, 1 or 0 mg elastin-orcein and incubated at 37 °C for 15 min with stirring. The samples were centrifuged for 10 min at 18,000× *g* and 4 °C. The supernatants were removed for SDS-PAGE analysis; the pellets were washed twice with Buffer A, resuspended with an equivalent volume of 10% SDS and boiled for 10 min to release the binding protein from elastin-orcein. The protein in the supernatant and the protein released from elastin-orcein were analysed by SDS-PAGE. Enzyme mixed with 0 mg elastin-orcein or BSA (0.2 mg/mL) in place of myroilysin was used as the controls. Quantitative analyses of the protein bands in the gel were densitometrically carried out using the program Quantity One Version 4.5 (Bio-Rad, Hercules, CA, USA).

To further confirm the ability of myroilysin to bind to elastin-orcein, the elastin-degrading activity of myroilysin bound to elastin-orcein was measured. Myroilysin (0.1 mg/mL, 0.25 mL) inactivated in Buffer A was mixed with 5 mg elastin-orcein, as described above. Elastin-orcein (5 mg) mixed with 0.25 mL Tris-HCl (50 mM, pH 9.0) served as a control. After washing twice, the precipitate of elastin-orcein with bound myroilysin was resuspended in 50 mM Tris-HCl (pH 9.0), incubated at 4 °C for 60 min and then centrifuged. This step was repeated to ensure that the residual Zn^2+^ was removed and that the activity of myroilysin to elastin-orcein was recovered. Then, the elastin-degrading activity of myroilysin bound to elastin-orcein was measured as described previously [3]. Briefly, elastin-orcein with bound myroilysin was resuspended in 0.25 mL 50 mM Tris-HCl (pH 9.0) and then incubated at 37 °C for 1 h with continuous stirring. The residual elastin-orcein was removed by centrifugation, and the absorption of the supernatant at 590 nm was measured. One unit of enzyme activity was defined as the amount of enzyme that caused an increase in 0.01 unit of absorbance at 590 nm per min.

### 4.3. Effects of NaCl and Nonionic Detergents on the Binding of Myroilysin to Insoluble Elastin

Myroilysin (0.3 mg/mL, 0.2 mL) inactivated in Buffer A was mixed with 8 mg elastin-orcein and incubated at 37 °C for 30 min with stirring. After incubation, the mixture was centrifuged for 10 min at 18,000× *g* and 4 °C. The resulting precipitate was washed twice in Buffer A and resuspended in 80 μL Tris-HCl (50 mM, pH 9.0) containing NaCl (2.0 M, 1.0 M or 0.5 M) or nonionic detergent (1% Tween 20, 1% Tween 60 or 1% Triton X-100, v/v). The precipitate resuspended in 80 μL Buffer A without any detergent or NaCl served as the controls. The mixtures were incubated for 60 min at 0 °C and then centrifuged. The precipitates were washed, and the bound myroilysin was released and subjected to SDS-PAGE analysis as described above. The protein bands in the SDS-PAGE gel were quantitatively analysed using the program Quantity One Version 4.5 (Bio-Rad, Hercules, CA, USA).

### 4.4. LM In Situ Observation of Bovine Elastin Fibre Degradation by Myroilysin

A trace amount of bovine elastin powders was placed on a glass slide and dipped in a drop of a solution containing 0.3 mg myroilysin in 1 mL 50 mM Tris-HCl buffer (pH 9.0). The drop was covered with a coverslip slowly from one side to avoid bubble formation. The prepared samples were immediately observed under an inverted microscope (Olympus IX71, Tokyo, Japan) at room temperature.

### 4.5. SEM Observation of Bovine Elastin Fibre Degradation by Myroilysin

Elastic fibres before and after enzymatic treatment were observed by SEM. A mixture of 0.2 mL myroilysin (0.05 mg/mL) with 5 mg bovine elastin fibres in 50 mM Tris-HCl buffer (pH 9.0) was incubated at 37 °C with continuous stirring. The same mixture containing no myroilysin was used as a control. After the reaction was stopped by the addition of trifluoroacetic acid to a final concentration of 1.0%, the residual material was separated by settling for 60 min and then washed twice with deionized water. The samples were lyophilized, mounted on a metal stub and sputter-coated with 5 nm platinum prior to examination with a Hitachi FE-S4800SEM (Hitachi, Tokyo, Japan) at 5.0 kV.

### 4.6. SEM Observation of the Degradation of Cross-Linked Recombinant Tropoelastin by Myroilysin

Recombinant tropoelastin was purified and cross-linked as previously described [14]. The cross-linked tropoelastin (100 μg) was mixed with myroilysin (2 μg) in 30 μL of 50 mM Tris-HCl buffer, pH 9.0. The mixture was incubated at 37 °C for 20, 60 or 150 min. After washing with cold 10 mM phosphate-buffered saline (pH 7.4), the samples were dehydrated with increasing concentrations of ethanol and sputter-coated for examination by SEM [14].

### 4.7. Analysis of Myroilysin Cleavage Sites in Bovine Elastin and Recombinant Human Tropoelastin

Bovine elastin (30 mg) was digested overnight with 0.15 mg myroilysin in 50 mM Tris-HCl (pH 9.0) at 37 °C. Recombinant human tropoelastin (0.2 mg) was digested overnight with 0.01 mg myroilysin in 50 mM Tris-HCl (pH 9.0) at 37 °C. The samples were then boiled for 10 min to terminate the reaction and centrifuged for 20 min at 18,000× *g* and 4 °C. LC/MS analysis was used to separate the released peptides in the supernatant and to determine the molecular masses of the peptides. The high resolution LC/MS analysis was performed on a LTQ Orbitrap Velos pro ETD mass spectrometer (Thermo Scientific, Waltham, MA, USA) equipped with a Prominence nano LC system (SHIMADZU, Kyoto, Japan) and a nanospray ionization source (NSI) in the positive mode. Sequences were determined using MASCOT MS/MS Ion Research tools (Version 2.4.0, Matrix Science, London, UK) in the Decoy database. The International Protein Index (IPI) was used. The database was taxonomically restricted to “Bos taurus” for bovine elastin hydrolysate analysis and to “Homo sapiens” for recombinant human tropoelastin hydrolysate analysis. “Oxidation (P)” was considered as a variable modification. The mass error tolerances for the peptides were typically set to 10 ppm. The significance threshold *p* was set to 0.02, and the peptides with an expect value less than 0.05 were selected.

## 5. Conclusions

This article studied the elastin degradation mechanism of the marine bacterial elastase myroilysin by biochemical analysis and microscopic observation. Myroilysin hydrophobically binds to the hydrophobic domains on the surface of an elastin fibre and preferentially cleaves peptide bonds of elastin with hydrophobic residues at the P1 and/or P1′ positions. The degradation leads to the formation of crevices and cavities on the fibre surface in the beginning and a final deformation of the fibre by stepwise fragmentation. Our results indicate that myroilysin degrades elastin by a mechanism different from pseudoalterin or MMPs. This study lays a foundation for developing the biotechnological application of myroilysin.

## Supplementary Files

Supplementary File 1
