# Supplementary material for: Mechanistic Insight into the Elastin Degradation Process by the Metalloprotease Myroilysin from the Deep-Sea Bacterium Myroides profundi D25
Source: Mar Drugs. 2015 Mar 18;13(3):1481–96. doi: 10.3390/md13031481 (PMC4377995; doi:10.3390/md13031481)
Supplement: Supplementary File 1 [file marinedrugs-13-01481-s001.pdf]

Supplementary Information

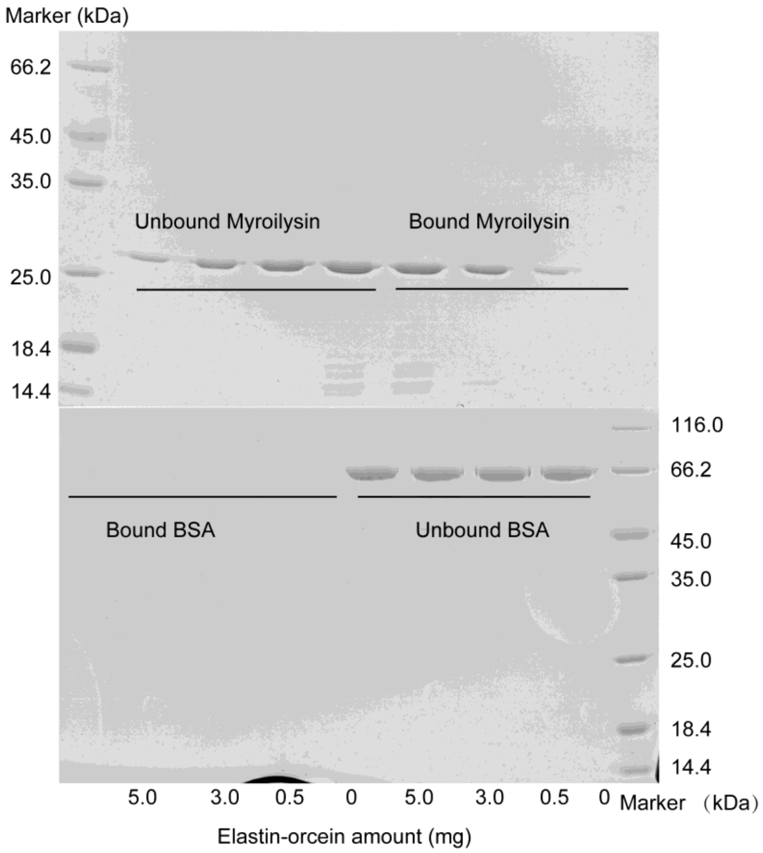

Figure S1. SDS-PAGE analysis of the binding ability of myroilysin to insoluble elastin. BSA was used as a negative control. The unbound and bound fractions were analyzyed by 12.5% SDS-PAGE. For protein staining, the gels were stained with 1.0% (wt/vol) Coomassie brilliant blue R-250. (Full-length gels of Figure 1A).

Table S1. The determined molecular masses and sequences of the 93 peptides released from bovine elastin fibers by myroilysin.

Number	Position	Sequence Identified [a]	Score	Expect Value	Error (ppm)	*m/z*	Mr (expt) (Da)	Mr (calc) (Da)
1	33–52	A.VPGGVPGGVFFPGAGLGGLG.V	42.68	0.002	−0.41	856.4616	1710.9087	1710.9094
2	33–54	A.VPGGVPGGVFFPGAGLGGLGVG.G	30.06	0.0084	0.11	934.507	1866.9995	1866.9993
3	37–52	G.VPGGVFFPGAGLGGLG.V	49.13	0.00056	−0.14	701.3798	1400.7451	1400.7453
4	37–54	G.VPGGVFFPGAGLGGLGVG.G	48.47	0.0009	−0.26	779.4247	1556.8348	1556.8352
5	40–52	G.GVFFPGAGLGGLG.V	49.52	0.00084	−0.87	574.8081	1147.6017	1147.6026
6	40–49	G.GVFFPGAGLG.G	46.45	0.023	1.85	461.2459	920.4773	920.4756
7	41–52	G.VFFPGAGLGGLG.V	58.45	4.90×10^{5}	−0.28	546.2977	1090.5809	1090.5812
8	41–50	G.VFFPGAGLGG.L	51.9	0.0008	0.76	461.2454	920.4763	920.4756
9	41–49	G.VFFPGAGLG.G	57.75	0.0012	0.00	432.7343	863.4541	863.4542
10	41–51	G.VFFPGAGLGGL.G	42.54	0.0065	0.97	517.7876	1033.5606	1033.5597
11	42–52	V.FFPGAGLGGLG.V	55.84	0.00037	−0.91	496.7632	991.5118	991.5127
12	112–127	G.AAGLGVGGIGGVGGLG.V	45.31	0.037	−2.06	606.3395	1210.6645	1210.667
13	113–129	A.AGLGVGGIGGVGGLGVS.T	28.34	0.035	−0.38	663.8722	1325.7298	1325.7304
14	114–127	A.GLGVGGIGGVGGLG.V	74.1	3.30E-05	−0.09	535.3036	1068.5927	1068.5928
15	115–127	G.LGVGGIGGVGGLG.V	70.19	7.40E-05	−0.30	506.7928	1011.571	1011.5713
16	115–129	G.LGVGGIGGVGGLGVS.T	49.79	0.0013	−2.25	599.8418	1197.669	1197.6718
17	115–126	G.LGVGGIGGVGGL.G	52.13	0.0041	0.94	478.2826	954.5507	954.5499
18	117–128	G.VGGIGGVGGLGV.S	65.07	0.00023	−0.11	471.2744	940.5341	940.5342
19	117–127	G.VGGIGGVGGLG.V	64.13	0.00042	0.12	421.7402	841.4659	841.4658
20	117–129	G.VGGIGGVGGLGVS.T	64.29	0.00043	0.00	514.7904	1027.5663	1027.5663
21	117–132	G.VGGIGGVGGLGVSTGA.V	46.05	0.0014	1.11	629.3442	1256.6739	1256.6725
22	117–137	G.VGGIGGVGGLGVSTGAVVPQL.G	22.81	0.029	−0.28	897.5094	1793.0042	1793.0048
23	120–132	G.IGGVGGLGVSTGA.V	60.98	0.00056	−0.38	522.7877	1043.5608	1043.5612
24	120–131	G.IGGVGGLGVSTG.A	35.2	0.013	0.10	487.2693	972.5241	972.524
25	120–130	G.IGGVGGLGVST.G	44.28	0.034	0.44	458.7588	915.503	915.5026
26	153–170	G.VGLPGVYPGGVLPGAGAR.F	27.65	0.037	−0.73	826.959	1651.9034	1651.9046
27	155–178	G.LPGVYPGGVLPGAGARFPGIGVLP.G	31.29	0.0094	−0.53	1131.143	2260.2721	2260.2732
28	162–178	G.GVLPGAGARFPGIGVLP.G	53.11	0.0032	−0.44	789.4614	1576.9083	1576.909

Table S1. *Cont.*

29	163–173	G.VLPGAGARFPG.I	47.9	0.0018	−0.19	521.2955	1040.5765	1040.5767
30	163–178	G.VLPGAGARFPGIGVLP.G	36.78	0.043	−0.26	760.9508	1519.8871	1519.8875
31	168–183	A.GARFPGIGVLPGVPTG.A	47.09	0.0075	1.34	747.926	1493.8375	1493.8355
32	168–184	A.GARFPGIGVLPGVPTGA.G	42.85	0.011	−1.47	783.4424	1564.8703	1564.8726
33	168–177	A.GARFPGIGVL.P	43.23	0.046	0.71	493.7931	985.5716	985.5709
34	168–178	A.GARFPGIGVLP.G	41.37	0.05	0.09	542.3192	1082.6237	1082.6237
35	339–368	G.VPGVGVPGVGVPGVGVPGVGVPGVGVPGVG.V	48.68	0.011	−0.12	825.4758	2473.4055	2473.4058
36	344–373	G.VPGVGVPGVGVPGVGVPGVGVPGVGVPGVG.V	48.68	0.011	−0.12	825.4758	2473.4055	2473.4058
37	349–378	G.VPGVGVPGVGVPGVGVPGVGVPGVGVPGVG.V	48.68	0.011	−0.12	825.4758	2473.4055	2473.4058
38	354–383	G.VPGVGVPGVGVPGVGVPGVGVPGVGVPGVG.V	48.68	0.011	−0.12	825.4758	2473.4055	2473.4058
39	359–388	G.VPGVGVPGVGVPGVGVPGVGVPGVGVPGVG.V	48.68	0.011	−0.12	825.4758	2473.4055	2473.4058
40	339–353	G.VPGVGVPGVGVPGVG.V	35.47	0.018	−2.25	623.86	1245.7054	1245.7082
41	344–358	G.VPGVGVPGVGVPGVG.V	35.47	0.018	−2.25	623.86	1245.7054	1245.7082
42	349–363	G.VPGVGVPGVGVPGVG.V	35.47	0.018	−2.25	623.86	1245.7054	1245.7082
43	354–368	G.VPGVGVPGVGVPGVG.V	35.47	0.018	−2.25	623.86	1245.7054	1245.7082
44	359–373	G.VPGVGVPGVGVPGVG.V	35.47	0.018	−2.25	623.86	1245.7054	1245.7082
45	364–378	G.VPGVGVPGVGVPGVG.V	35.47	0.018	−2.25	623.86	1245.7054	1245.7082
46	369–383	G.VPGVGVPGVGVPGVG.V	35.47	0.018	−2.25	623.86	1245.7054	1245.7082
47	374–388	G.VPGVGVPGVGVPGVG.V	35.47	0.018	−2.25	623.86	1245.7054	1245.7082
48	339–361	G.VPGVGVPGVGVPGVGVPGVGVPG.V	18.2	0.026	−1.42	637.0342	1908.0807	1908.0834
49	344–366	G.VPGVGVPGVGVPGVGVPGVGVPG.V	18.2	0.026	−1.42	637.0342	1908.0807	1908.0834
50	349–371	G.VPGVGVPGVGVPGVGVPGVGVPG.V	18.2	0.026	−1.42	637.0342	1908.0807	1908.0834
51	354–376	G.VPGVGVPGVGVPGVGVPGVGVPG.V	18.2	0.026	−1.42	637.0342	1908.0807	1908.0834
52	359–381	G.VPGVGVPGVGVPGVGVPGVGVPG.V	18.2	0.026	−1.42	637.0342	1908.0807	1908.0834
53	364–386	G.VPGVGVPGVGVPGVGVPGVGVPG.V	18.2	0.026	−1.42	637.0342	1908.0807	1908.0834
54	369–391	G.VPGVGVPGVGVPGVGVPGVGVPG.V	18.2	0.026	−1.42	637.0342	1908.0807	1908.0834
55	339–363	G.VPGVGVPGVGVPGVGVPGVGVPGVG.V	42.83	0.045	−0.78	689.0645	2064.1717	2064.1733
56	344–368	G.VPGVGVPGVGVPGVGVPGVGVPGVG.V	42.83	0.045	−0.78	689.0645	2064.1717	2064.1733
57	349–373	G.VPGVGVPGVGVPGVGVPGVGVPGVG.V	42.83	0.045	−0.78	689.0645	2064.1717	2064.1733

Table S1. *Cont.*

58	354–378	G.VPGVGVPGVGVPGVGVPGVGVPGVG.V	42.83	0.045	−0.78	689.0645	2064.1717	2064.1733
59	359–383	G.VPGVGVPGVGVPGVGVPGVGVPGVG.V	42.83	0.045	−0.78	689.0645	2064.1717	2064.1733
60	364–388	G.VPGVGVPGVGVPGVGVPGVGVPGVG.V	42.83	0.045	−0.78	689.0645	2064.1717	2064.1733
61	344–392	G.VPGVGVPGVGVPGVGVPGVGVPGVGVPGVGVPGVGVPGVGVP GVGVPGA.L	43.18	0.002	0.67	806.0644	4025.2859	4025.2832
62	364–392	G.VPGVGVPGVGVPGVGVPGVGVPGVGVPGA.L	46.51	0.00071	0.29	797.1252	2388.3537	2388.353
63	496–521	A.QFGLGPGVGVAPGVGVVPGVGVVPGV.G	28.79	0.018	−2.33	1136.144	2270.2735	2270.2788
64	498–521	F.GLGPGVGVAPGVGVVPGVGVVPGV.G	55.89	3.50E-05	0.15	666.058	1995.1521	1995.1518
65	498–526	F.GLGPGVGVAPGVGVVPGVGVVPGVGVAPG.I	61.29	4.50E-05	−0.08	793.1249	2376.3528	2376.353
66	498–519	F.GLGPGVGVAPGVGVVPGVGVVP.G	39.16	0.0015	0.76	920.5389	1839.0633	1839.0619
67	498–510	F.GLGPGVGVAPGVG.V	45.09	0.0051	0.10	518.793	1035.5714	1035.5713
68	498–538	F.GLGPGVGVAPGVGVVPGVGVVPGVGVAPGIGLGPGGVIGAG.V	36.7	0.017	0.24	665.9859	3324.8931	3324.8923
69	498–508	F.GLGPGVGVAPG.V	43.13	0.034	−0.23	440.7479	879.4813	879.4814
70	499–526	G.LGPGVGVAPGVGVVPGVGVVPGVGVAPG.I	29.03	0.019	−0.86	774.1171	2319.3296	2319.3316
71	505–521	G.VAPGVGVVPGVGVVPGV.G	31.58	0.016	−0.07	729.9376	1457.8606	1457.8607
72	508–521	P.GVGVVPGVGVVPGV.G	47.77	0.0074	−0.34	596.3583	1190.702	1190.7024
73	509–521	G.VGVVPGVGVVPGV.G	49.42	0.00071	−0.44	567.8475	1133.6804	1133.6809
74	509–526	G.VGVVPGVGVVPGVGVAPG.I	42.84	0.0011	−0.20	758.4482	1514.8818	1514.8821
75	509–522	G.VGVVPGVGVVPGVG.V	46.94	0.0013	0.08	596.3585	1190.7025	1190.7024
76	509–533	G.VGVVPGVGVVPGVGVAPGIGLGPGG.V	38.43	0.0013	−1.89	689.7356	2066.185	2066.1889
77	509–536	G.VGVVPGVGVVPGVGVAPGIGLGPGGVIG.A	26.75	0.0035	−1.03	779.4607	2335.3604	2335.3629
78	511–538	G.VVPGVGVVPGVGVAPGIGLGPGGVIGAG.V	31.23	0.0025	−0.30	770.1176	2307.3308	2307.3315
79	511–521	G.VVPGVGVVPGV.G	27.89	0.026	−0.10	489.8027	977.5909	977.591
80	521–533	G.VGVAPGIGLGPGG.V	66.6	7.90E-05	0.67	525.8011	1049.5876	1049.587
81	521–538	G.VGVAPGIGLGPGGVIGAG.V	26.69	0.037	−2.21	724.4154	1446.8163	1446.8195
82	522–538	V.GVAPGIGLGPGGVIGAG.V	47.33	0.0012	0.30	674.883	1347.7514	1347.7511
83	526–538	P.GIGLGPGGVIGAG.V	83.26	3.20E–06	−0.29	512.7928	1023.571	1023.5713
84	527–537	G.IGLGPGGVIGA.G	61.98	0.00024	0.44	455.7717	909.5288	909.5284
85	527–536	G.IGLGPGGVIG.A	54.28	0.00044	0.12	420.253	838.4914	838.4913

Table S1. *Cont*.

86	527–538	G.IGLGPGGVIGAG.V	53.34	0.0023	0.52	484.2825	966.5504	966.5498
87	651–667	A.AQFGLGGVGGLGVGGLG.A	42.13	0.0052	–0.99	708.385	1414.7555	1414.7569
88	652–667	A.QFGLGGVGGLGVGGLG.A	56.99	0.00075	−1.27	672.8663	1343.7181	1343.7198
89	653–662	Q.FGLGGVGGLG.V	44.21	0.044	−0.48	417.2292	832.4439	832.4443
90	654–667	F.GLGGVGGLGVGGLG.A	91.48	7.60E-07	0.66	535.304	1068.5935	1068.5928
91	654–668	F.GLGGVGGLGVGGLGA.V	71.86	3.40E-05	0.18	570.8223	1139.6301	1139.6299
92	655–667	G.LGGVGGLGVGGLG.A	83.83	3.00E-06	0.49	506.7932	1011.5719	1011.5713
93	694–707	G.LGGVLGAGQPFPIG.G	29.88	0.02	−1.09	641.8607	1281.7068	1281.7081

[a] The sequence of each peptide was determined by high resolution LC/MS analysis and MASCOT MS/MS Ion Research tools. The sequences in red are those appearing repeatly in bovine tropoelastin.

Table S2. The determined molecular masses and sequences of the 88 peptides released from recombinant human tropoelastin by myroilysin.

Number	Position	Sequence Identified [b]	Score	Expect Value	Error (ppm)	*m/z*	Mr (expt) (Da)	Mr (calc) (Da)
1	33–69	A.IPGGVPGGVFYPGAGLGALGGGALGPGGKPLKPVPGG.L	33.72	0.00076	–0.41	1065.927	3194.7592	3194.7605
2	51–69	A.LGGGALGPGGKPLKPVPGG.L	61.3	7.40E-07	–0.86	814.977	1627.9395	1627.941
3	51–65	A.LGGGALGPGGKPLKP.V	49.7	1.10E-05	–1.90	659.8945	1317.7744	1317.7769
4	51–68	A.LGGGALGPGGKPLKPVPG.G	32.75	0.00053	–0.06	786.467	1570.9194	1570.9195
5	51–67	A.LGGGALGPGGKPLKPVP.G	31.03	0.00079	–2.97	757.954	1513.8935	1513.8981
6	52–67	L.GGGALGPGGKPLKPVP.G	28.93	0.0013	–1.71	701.4131	1400.8116	1400.814
7	52–69	L.GGGALGPGGKPLKPVPGG.L	28.46	0.0023	0.92	758.4365	1514.8584	1514.8569
8	52–65	L.GGGALGPGGKPLKP.V	23.14	0.0049	–1.83	402.5708	1204.6906	1204.6928
9	53–65	G.GGALGPGGKPLKP.V	55.73	2.70E-06	0.09	574.843	1147.6715	1147.6713
10	53–67	G.GGALGPGGKPLKPVP.G	52.27	5.90E-06	–1.41	672.9026	1343.7906	1343.7925
11	53–69	G.GGALGPGGKPLKPVPGG.L	51.17	7.60E-06	–1.37	729.924	1457.8335	1457.8355
12	53–62	G.GGALGPGGKP.L	38.97	0.00013	–0.37	405.7269	809.4392	809.4396
13	53–63	G.GGALGPGGKPL.K	36.36	0.00023	–0.65	462.2688	922.523	922.5236
14	53–68	G.GGALGPGGKPLKPVPG.G	26.17	0.0024	–0.43	467.9451	1400.8134	1400.814
15	53–64	G.GGALGPGGKPLK.P	17.63	0.017	–0.10	526.3165	1050.6185	1050.6186
16	54–69	G.GALGPGGKPLKPVPGG.L	52.61	5.50E-06	–0.50	701.4139	1400.8133	1400.814
17	54–67	G.GALGPGGKPLKPVP.G	33.09	0.00049	–1.32	644.392	1286.7694	1286.7711
18	54–65	G.GALGPGGKPLKP.V	31.92	0.00064	–2.38	546.3309	1090.6473	1090.6499
19	54–63	G.GALGPGGKPL.K	24.81	0.0033	0.12	433.7584	865.5023	865.5022
20	55–65	G.ALGPGGKPLKP.V	52.66	5.40E-06	–1.06	517.8209	1033.6273	1033.6284
21	55–68	G.ALGPGGKPLKPVPG.G	52.02	6.30E-06	–1.40	644.3919	1286.7693	1286.7711
22	55–67	G.ALGPGGKPLKPVP.G	41.01	7.90E-05	–2.68	615.8804	1229.7463	1229.7496
23	55–63	G.ALGPGGKPL.K	38.12	0.00015	–1.24	405.2471	808.4796	808.4807
24	55–69	G.ALGPGGKPLKPVPGG.L	18.44	0.014	–0.15	448.938	1343.7923	1343.7925
25	56–65	A.LGPGGKPLKP.V	42.72	5.30E-05	0.10	482.303	962.5914	962.5913
26	56–69	A.LGPGGKPLKPVPGG.L	41.79	6.60E-05	–1.57	637.384	1272.7534	1272.7554
27	56–67	A.LGPGGKPLKPVP.G	37.22	0.00019	–1.81	580.3625	1158.7104	1158.7125
28	56–63	A.LGPGGKPL.K	17.12	0.019	0.41	369.7292	737.4438	737.4436

Table S2. *Cont.*

29	57–69	L.GPGGKPLKPVPGG.L	49.02	1.30E-05	–0.95	580.8424	1159.6703	1159.6714
30	57–67	L.GPGGKPLKPVP.G	42.55	5.60E-05	0.57	523.8218	1045.629	1045.6284
31	57–65	L.GPGGKPLKP.V	39.87	0.0001	0.47	425.7611	849.5077	849.5072
32	57–68	L.GPGGKPLKPVPG.G	18.29	0.015	–1.09	368.5569	1102.6487	1102.6499
33	58–67	G.PGGKPLKPVP.G	34.66	0.00034	0.10	495.3108	988.607	988.6069
34	58–69	G.PGGKPLKPVPGG.L	30.68	0.00086	–0.09	368.5572	1102.6497	1102.6499
35	58–65	G.PGGKPLKP.V	18.66	0.014	–0.13	397.2501	792.4856	792.4858
36	59–69	P.GGKPLKPVPGG.L	20.84	0.0082	–1.09	503.8053	1005.596	1005.5971
37	60–69	G.GKPLKPVPGG.L	23.31	0.0047	–2.95	475.2937	948.5728	948.5757
38	60–67	G.GKPLKPVP.G	13.95	0.04	–0.36	418.2735	834.5324	834.5327
39	61–67	G.KPLKPVP.G	37.17	0.00019	–0.77	389.7626	777.5106	777.5112
40	61–69	G.KPLKPVPGG.L	32.38	0.00058	–0.11	446.7843	891.554	891.5542
41	61–68	G.KPLKPVPG.G	26.97	0.002	0.36	418.2738	834.533	834.5327
42	104–157	Y.KAAKAGAGLGGVPGVGGLGVSAGAVVPQPGAGVKPGKVPGVGLP GVYPGGVLPG.A	16.54	0.043	0.36	676.9515	4731.6094	4731.6077
43	105–122	K.AAKAGAGLGGVPGVGGLG.V	29.8	0.0016	–1.56	704.8979	1407.7812	1407.7834
44	114–122	G.GVPGVGGLG.V	40.64	0.00017	0.42	356.7032	711.3918	711.3916
45	656–664	L.GVPGVGGLG.G	40.64	0.00017	0.42	356.7032	711.3918	711.3916
46	126–134	A.GAVVPQPGA.G	17.37	0.018	0.13	398.2217	794.4287	794.4287
47	128–166	A.VVPQPGAGVKPGKVPGVGLPGVYPGGVLPGARFPGVGVL.P	19.27	0.035	–3.47	1248.024	3741.0489	3741.0619
48	141–159	K.VPGVGLPGVYPGGVLPGAR.F	35.5	0.00051	–0.97	881.5033	1760.992	1760.9938
49	141–178	K.VPGVGLPGVYPGGVLPGARFPGVGVLPGVPTGAGVKPK.A	27.25	0.002	–0.74	880.7599	3519.0105	3519.013
50	146–153	G.LPGVYPGG.V	35.07	0.00047	–0.79	380.2051	758.3957	758.3963
51	146–159	G.LPGVYPGGVLPGAR.F	21.81	0.0066	0.52	676.8882	1351.7619	1351.7612
52	148–156	P.GVYPGGVLP.G	16.82	0.033	–0.23	429.7395	857.4645	857.4647
53	157–178	P.GARFPGVGVLPGVPTGAGVKPK.A	15.29	0.03	1.26	516.0553	2060.1921	2060.1895
54	160–178	R.FPGVGVLPGVPTGAGVKPK.A	45.62	2.70E-05	–0.45	889.0218	1776.029	1776.0298
55	188–196	A.GIPGVGPFG.G	27.31	0.0021	–0.13	400.7187	799.4228	799.4229
56	189–196	G.IPGVGPFG.G	24.02	0.0048	–1.08	372.2076	742.4006	742.4014

Table S2. *Cont.*

57	206–212	G.YPIKAPK.L	14.61	0.035	0.12	408.7526	815.4906	815.4905
58	210–218	K.APKLPGGYG.L	35.13	0.00048	0.23	430.2374	858.4602	858.4599
59	213–222	K.LPGGYGLPYT.T	15.37	0.032	–2.00	527.2651	1052.5157	1052.5179
60	242–254	K.AGYPTGTGVGPQA.A	40.41	0.00013	–0.85	588.2877	1174.5609	1174.5619
61	289–303	A.IPGIGGIAGVGTPAA.A	33.43	0.00045	–0.32	625.8586	1249.7026	1249.703
62	321–329	A.GLVPGGPGF.G	40.32	0.00011	–0.25	400.7186	799.4226	799.4229
63	322–329	G.LVPGGPGF.G	53	6.00E-06	–0.40	372.2078	742.4011	742.4014
64	350–357	A.GIPVVPGA.G	34.77	0.00033	–0.56	355.2156	708.4167	708.417
65	350–358	A.GIPVVPGAG.I	26.09	0.0036	–0.13	383.7265	765.4384	765.4385
66	351–358	G.IPVVPGAG.I	35.6	0.00028	–1.98	355.2151	708.4156	708.417
67	364–372	A.VPGVVSPEA.A	14.15	0.044	0.12	427.7346	853.4547	853.4545
68	374–395	A.AKAAAKAAKYGARPGVGVGGIP.T	17.7	0.017	–0.44	507.2941	2025.1474	2025.1483
69	384–398	Y.GARPGVGVGGIPTYG.V	28.68	0.0026	–2.28	679.3632	1356.7119	1356.715
70	399–407	G.VGAGGFPGF.G	32.73	0.00053	0.50	404.7033	807.392	807.3916
71	399–419	G.VGAGGFPGFGVGVGGIPGVAG.V	22.47	0.014	–0.75	864.9564	1727.8983	1727.8996
72	472–482	F.GLVPGVGVAPG.V	29.64	0.0013	–0.54	461.7712	921.5279	921.5284
73	472–497	F.GLVPGVGVAPGVGVAPGVGVAPGVGL.A	25.33	0.0029	0.00	718.0878	2151.2417	2151.2417
74	473–481	G.LVPGVGVAP.G	20.23	0.0095	0.25	404.7501	807.4857	807.4855
75	480–489	V.APGVGVAPGV.G	19.73	0.02	–0.97	412.2369	822.4592	822.46
76	486–495	V.APGVGVAPGV.G	19.73	0.02	–0.97	412.2369	822.4592	822.46
77	498–507	L.APGVGVAPGV.G	19.73	0.02	–0.97	412.2369	822.4592	822.46
78	504–513	V.APGVGVAPGV.G	19.73	0.02	–0.97	412.2369	822.4592	822.46
79	495–511	G.VGLAPGVGVAPGVGVAP.G	19.57	0.011	–0.85	708.9135	1415.8125	1415.8137
80	498–526	L.APGVGVAPGVGVAPGVGVAPGIGPGGVAA.A	62.32	9.70E-07	–1.21	769.7646	2306.2719	2306.2747
81	597–625	G.AAVPGVLGGLGALGGVGIPGGVVGAGPAA.A	42.08	7.40E-05	0.04	771.1093	2310.3061	2310.306
82	679–695	G.AAGLGGVLGGAGQFPLG.G	16.55	0.031	–0.62	721.3931	1440.7716	1440.7725
83	697–703	G.VAARPGF.G	14.32	0.037	1.40	359.2063	716.398	716.397
84	704–713	F.GLSPIFPGGA.C	53.48	5.40E-06	0.11	458.2504	914.4862	914.4862
85	704–712	F.GLSPIFPGG.A	41.47	0.00012	–0.24	422.7317	843.4489	843.4491

Table S2. *Cont.*

86	704–710	F.GLSPIFP.G	34.6	0.00068	0.27	365.7104	729.4063	729.4061
87	705–712	G.LSPIFPGG.A	43.41	6.80E-05	–0.89	394.2207	786.4269	786.4276
88	705–711	G.LSPIFPG.G	18.34	0.024	–0.14	365.7103	729.406	729.4061

[b] The sequence of each peptide was determined by high resolution LC/MS analysis and MASCOT MS/MS Ion Research tools. The sequences in red are those appearing repeatly in human tropoelastin.

© 2015 by the authors; licensee MDPI, Basel, Switzerland. This article is an open access article distributed under the terms and conditions of the Creative Commons Attribution license (http://creativecommons.org/licenses/by/4.0/).
